# Nonalcoholic Fatty Liver Disease and Omega-3 Fatty Acids: Mechanisms and Clinical Use

**DOI:** 10.1146/annurev-nutr-061021-030223

**Published:** 2023-05-19

**Authors:** Melinda H. Spooner, Donald B. Jump

**Affiliations:** Molecular Nutrition and Diabetes Research Laboratory, School of Biological and Population Health Sciences, Oregon State University, Corvallis, Oregon, USA

**Keywords:** docosahexaenoic acid, nonalcoholic steatohepatitis, steatosis, inflammation, fibrosis

## Abstract

Nonalcoholic fatty liver disease (NAFLD) is the most common chronic fatty liver disease worldwide, particularly in obese and type 2 diabetic individuals. Currently, there are no therapies for NAFLD that have been approved by the US Food and Drug Administration. Herein, we examine the rationale for using ω3 polyunsaturated fatty acids (PUFAs) in NAFLD therapy. This focus is based on the finding that NAFLD severity is associated with a reduction of hepatic C_20–22_ ω3 PUFAs. Because C_20–22_ ω3 PUFAs are pleiotropic regulators of cell function, loss of C_20–22_ ω3 PUFAs has the potential to significantly impact hepatic function. We describe NAFLD prevalence and pathophysiology as well as current NAFLD therapies. We also present evidence from clinical and preclinical studies that evaluated the capacity of C_20–22_ ω3 PUFAs to treat NAFLD. Given the clinical and preclinical evidence, dietary C_20–22_ ω3 PUFA supplementation has the potential to decrease human NAFLD severity by reducing hepatosteatosis and liver injury.

## INTRODUCTION

Nonalcoholic fatty liver disease (NAFLD) is the most common chronic fatty liver disease worldwide. While fatty liver is associated with many forms of malnutrition, such as kwashiorkor, Wilson’s disease, choline deficiency, and others (11, 27, 89, 134), we focus on NAFLD in the context of obesity, type 2 diabetes mellitus (T2DM), and metabolic syndrome (MetS). Nonalcoholic steatohepatitis (NASH), the progressive form of NAFLD, was first described in 1980 by Ludwig et al. (84) as a previously unknown liver disease that mimicked alcoholic hepatitis. Histological analysis of livers revealed lobular hepatitis, inflammatory infiltrates, focal necrosis, and evidence of liver injury. The disease was reported to be more common in women and associated with hepatomegaly, T2DM, and cholelithiasis, that is, gall stones.

**Figure 1:**
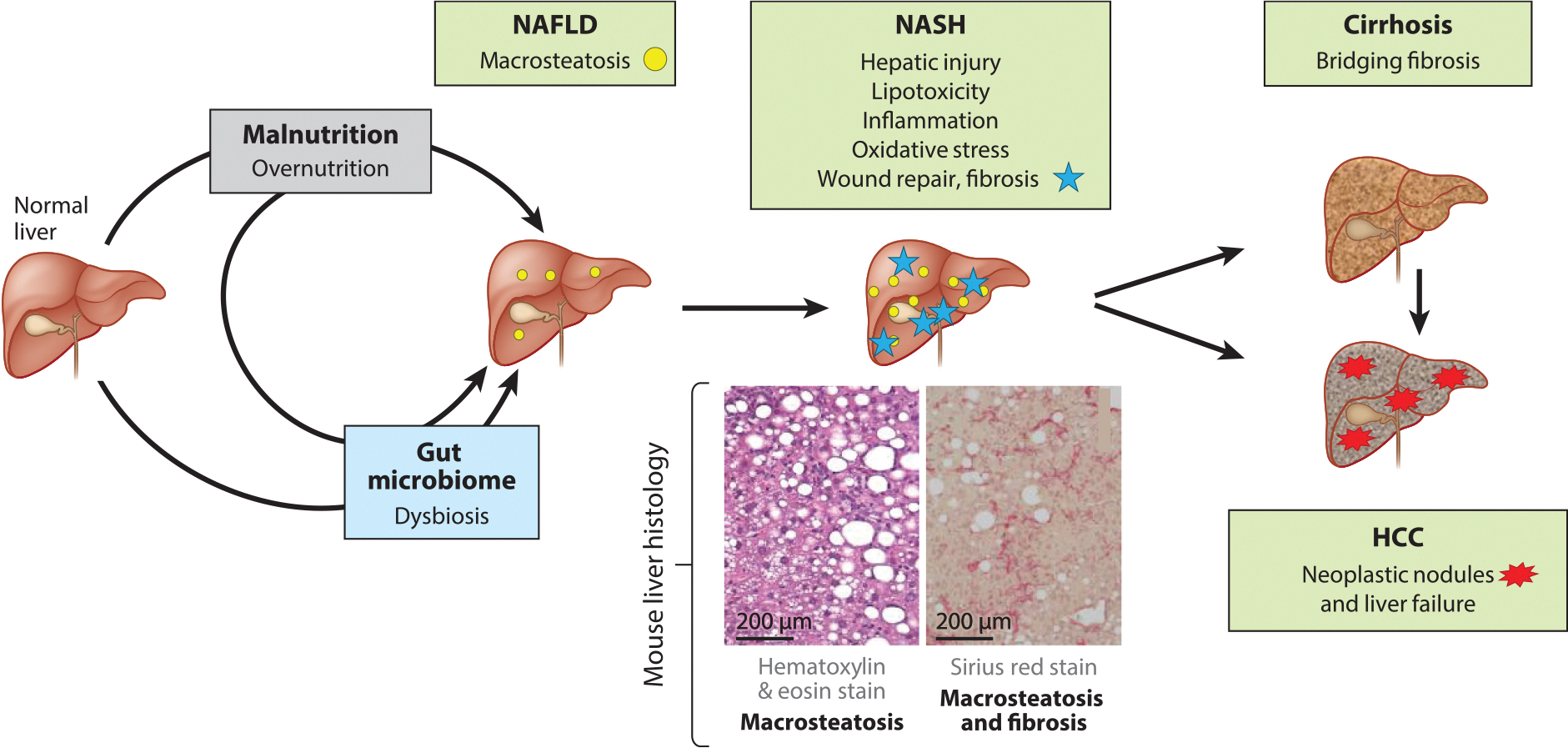
Diet and gut dysbiosis play a role in the transition of normal liver to hepatocellular cancer. Insets show mouse liver histology from western diet–fed *Ldlr*^−*/*−^ mice with NASH. Abbreviations: HCC, hepatocellular cancer; NAFLD, nonalcoholic fatty liver disease; NASH, nonalcoholic steatohepatitis.

NAFLD is the most common chronic liver disease associated with the accumulation of lipid in the liver. Since its initial description, research on NAFLD and NASH has grown significantly, with more than 30,000 citations contained in the PubMed database and more than 1,100 clinical trials listed in the ClinicalTrials.gov database (https://www.clinicaltrials.gov). NAFLD is a spectrum of liver diseases ranging from hepatic macrosteatosis (fatty liver) as defined by an aberrant accumulation of neutral lipids [triacylglycerols (TAGs), diacylglycerols (DAGs), and cholesterol esters (CEs)] in the liver to NASH (hepatic inflammation with liver injury), cirrhosis (extensive fibrosis), and hepatocellular cancer (HCC) (Figure 1). Recent evidence suggests that NASH can progress directly to HCC without first developing into cirrhosis (110, 129).

The earliest clinical evidence of NAFLD is the accumulation of neutral lipids in the liver, appearing as small lipid droplets [microvesicular steatosis (MIS)] and large lipid droplets [macrovesicular steatosis (MAS)] (130) (Figure 1). NAFLD, however, is typically associated with MAS. In addition to TAG and CE storage, excessive accumulation of free cholesterol in liver membranes and lipid droplets also plays a causal role in hepatic damage leading to NASH (51, 53, 91). Excessive accumulation of nonesterified fatty acids (NEFAs) and/or cholesterol in cells is toxic, causing liver injury, that is, hepatocellular death. The abnormal accumulation of lipids, appearing as MAS, NEFAs, or free cholesterol, is considered the first hit in a multihit, multicellular process leading to hepatic lipotoxicity, inflammation, oxidative stress, and hepatocellular injury. Hepatocellular death, arising from NASH, promotes tissue repair involving extracellular matrix (ECM) remodeling and the deposition of proteins associated with fibrosis, including collagens (Col1a1 and Col1a2), smooth muscle actin (Acta2), elastin (Eln), and ECM remodeling proteins, such as metalloproteases (MMPs), tissue inhibitors of metalloproteases (TIMPs), and lysyl oxidases (Lox and LoxL) involved in collagen cross-linking.

The accumulation of lipid and fibrosis within the liver can be assessed using noninvasive approaches such as ultrasound and magnetic resonance imaging (MRI) to quantify liver size and fat content and transient elastography to measure liver stiffness resulting from fibrosis. Hepatic injury is also assessed indirectly by measuring blood levels of enzymes, such as alanine amino transferase (ALT), aspartate amino transferase (AST), and γ-glutamyl transferase (GGT). However, the gold standard for NAFLD diagnosis involves histology of biopsied liver samples. This method provides details on hepatosteatosis (MIS versus MAS), hepatocyte ballooning (enlargement of hepatocytes by 1.5–2.0 times), necrosis, inflammatory infiltrate (leukocytes), and location and extent of fibrosis. The appearance of hepatocyte ballooning indicates progression from macrosteatosis to NASH (83). Scores for steatosis (0–3), lobular inflammation (0–3), hepatocyte ballooning (0–2), and fibrosis (0, 1a, 1b, 1c, 2, and 3) contribute to the NAFLD activity score (16).

NAFLD is associated with MetS, and MetS is a cluster of conditions including hyperglycemia, excess body fat around the waist (obesity), dyslipidemia (blood levels of cholesterol and/or TAG), and high blood pressure. These features are risk factors for heart disease, stroke, and T2DM. MetS represents the predominate feature in 36–67% of NAFLD patients, while 88% of NASH patients have MetS (22, 148). The presence of MetS further increases the rate of histologically confirmed NASH by 40% and increases overall mortality from NAFLD (29). A total of 7–30% of NAFLD patients progress to NASH, and 20–30% of NASH patients develop cirrhosis that can progress to HCC and liver failure (22, 29, 58) (Figure 1). The projected rates of decompensated NASH (loss of liver function), cirrhosis, and HCC are expected to increase 168%, 137%, and 178%, respectively, by 2030 (29, 38). This trajectory paints a bleak picture in the absence of US Food and Drug Administration (FDA)-approved NAFLD therapies and the increasing rates of obesity worldwide (29, 38).

## NAFLD IS A GLOBAL PUBLIC HEALTH PROBLEM

NAFLD is the most prevalent form of chronic liver disease globally, with ~25% prevalence worldwide (29, 101, 122). The obesity epidemic, with more than 1.9 billion overweight adults reported by the World Health Organization in 2016, parallels the increased number of patients with NAFLD (5, 29, 59, 122). The National Health and Nutrition Examination Survey for the United States estimated that 39.8% of adults are obese (29). Obesity severity increases the incidence of NAFLD, ranging from 75% in overweight individuals to 90–95% in morbidly obese individuals (29, 122, 132, 137). Obesity, T2DM, MetS, and dyslipidemia are the top four risk factors for NAFLD, and these diseases are strongly associated with fatty liver in patients with a body mass index (BMI) > 30 (59, 122). The prevalence of NAFLD varies by country, ranging from ~13% in Africa to ~30% in the United States to ~42% in southeast Asia (80, 148). NAFLD is the fastest growing cause of HCC in both the United States and Europe (52, 129, 143).

Multiple factors contribute to the onset of NAFLD including gender, diet, lifestyle, genetic polymorphisms [e.g., patatin-like phospholipase containing domain 3 (*Pnpla3*)], ethnicity, and health status (4, 22, 37, 47, 59, 146). Both children and adults develop NAFLD and NASH. Interestingly, premenopausal women are less prone to developing NAFLD than are children, men, and postmenopausal women (50, 142). Dietary fat plays an important role in the onset and progression of NAFLD to NASH. A recent prospective cohort study with children and young adults revealed the impact of diet on liver disease. The Raine Study, including 985 participants ranging in age from 14 to 22, reported that individuals at increased risk of fatty liver disease in early adolescence were associated with high dietary intake of ω6 polyunsaturated fatty acids (PUFAs) and a high dietary ω6/ω3 PUFA ratio (140). Children who present with NAFLD have increased risk of further complications in adulthood, such as NASH, T2DM, cardiovascular disease, and renal disease (22). A common cause of NAFLD mortality is cardiovascular disease (22, 131).

Liver transplantation due to NASH progression to HCC has increased >7-fold between 2002 and 2016 in both the United States and Europe (29). Further, NAFLD is second to alcoholic liver disease for liver transplants. Liver transplants are correlated with the increasing number of obese patients in the United States (29, 65). The increased rates of obesity in children and adults paralleling the prevalence of NAFLD is a major public health concern warranting intensive investigation of its pathogenesis and the development of novel therapeutic strategies.

## WHY FOCUS ON C_18–22_ ω3 PUFAs AS AN NAFLD THERAPY?

The severity of NAFLD, that is, progression from macrosteatosis to NASH, cirrhosis, and HCC, is associated with a decline in hepatic C_18–22_ ω3 and ω6 PUFAs (2, 7, 17, 18). Because hepatic ω3 PUFA levels are significantly lower than ω6 PUFA levels, pathways associated with ω3 PUFA function may be affected more than pathways associated with ω6 PUFA function. Moreover, this change in hepatic PUFA content increases the ω6/ω3 PUFA ratio, a ratio that is associated with increased inflammation (119). As such, NAFLD might be viewed as a ω3 PUFA deficiency disease, at least at the hepatic level. We examine this issue in detail below.

**Figure 2:**
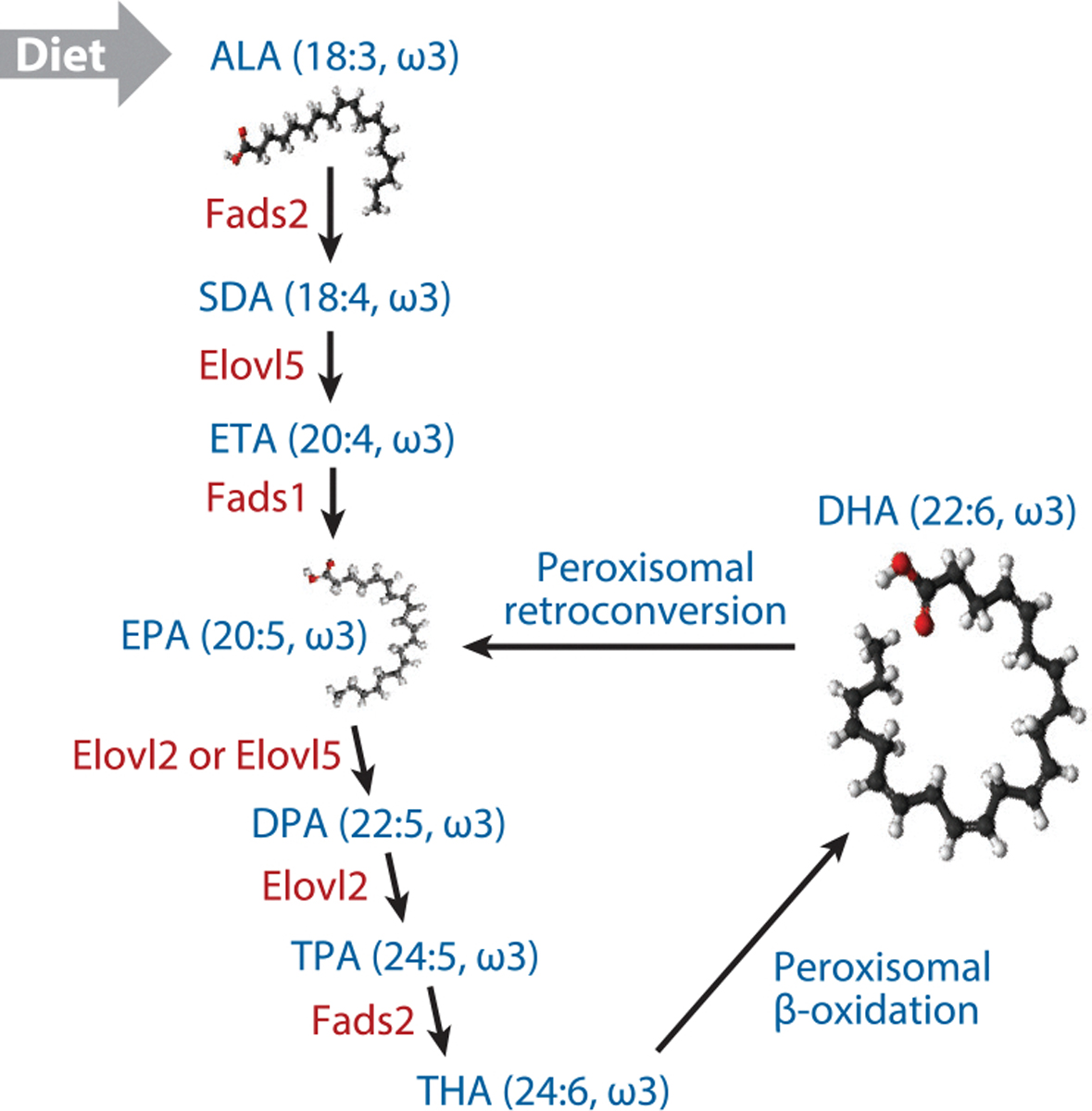
Pathway for the conversion of the essential fatty acid ALA to DHA. Abbreviations: ALA, α-linolenic acid; DHA, docosahexaenoic acid; DPA, docosapentaenoic acid; Elovl, fatty acid elongase; EPA, eicosapentaenoic acid; ETA, eicosatetraenoic acid; Fads, fatty acid desaturase; SDA, stearidonic acid; THA, tetracosahexaenoic acid; TPA, tetracosapentaenoic acid. Figure adapted from Reference 55.

α-Linolenic acid (ALA) (18:3, ω3) is an essential fatty acid and is the predominant ω3 fatty acid in the human diet. ALA undergoes a series of desaturation and elongation steps as well as peroxisomal β-oxidation to generate the end product of the pathway, that is, docosahexaenoic acid (DHA) (22:6, ω3) (Figure 2) (55). Humans, however, convert only a small fraction (0.2–9%) of dietary ALA to DHA (57), and DHA is the predominant ω3 PUFA accumulating in liver. While other hepatic ω3 PUFAs include stearidonic acid (SDA) (18:4, ω3), eicosapentaenoic acid (EPA) (20:5, ω3), and docosapentaenoic acid (DPA) (22:5, ω3), these fatty acids are present at low levels, relative to DHA. Interestingly, excess hepatic DHA is retroconverted to EPA and DPA through a peroxisomal chain-shortening process (60). In the sidebar titled Why Focus on ω3 PUFAs?, we list several reasons why dietary C_20–22_ ω3 PUFA supplementation has the potential to improve hepatic function.

**Figure 3:**
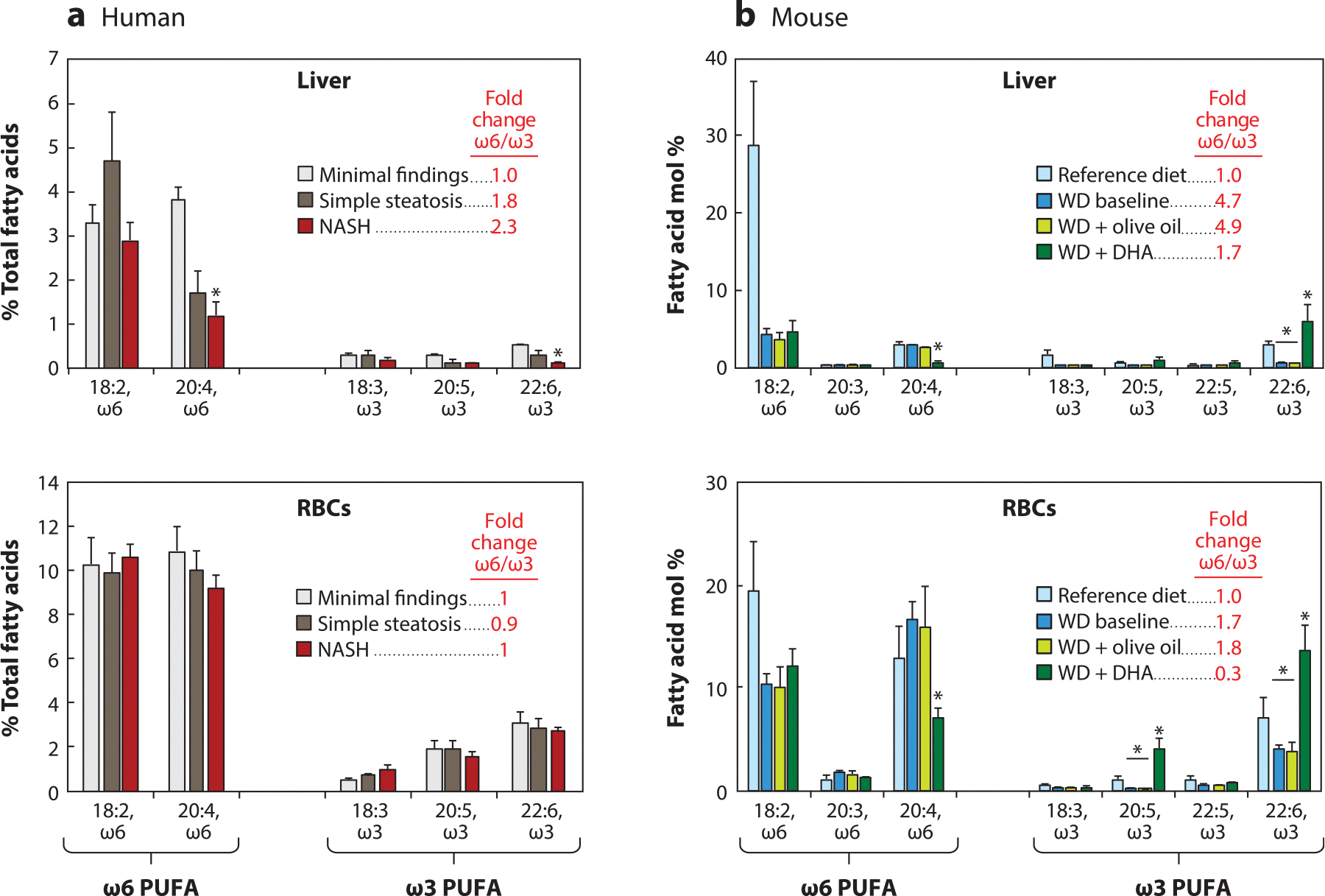
Assessment of ω6 and ω3 PUFAs in livers and RBCs of humans and mice. (*a*) ω6 and ω3 PUFAs in human liver and RBCs from patients with minimal features associated with NAFLD (minimal findings), simple steatosis, and NASH, as reported by Allard et al. (2). Results are presented as % total fatty acids, mean ± SEM. (*b*) ω6 and ω3 PUFAs in mouse liver and RBCs. Mouse liver data are from the report by Lytle et al. (87). The mouse RBC fatty acid data were not previously published but were obtained from the same animals as used for the liver data (K.A. Lytle & D.B. Jump, unpublished observations, 2016). Briefly, male *Ldlr*^−/−^ mice were maintained on a reference diet chow or a WD for 22 weeks. While the control mice were maintained on the reference diet until the end of the experiments, mice on the WD were switched to a WD supplemented with either olive oil or DHASCO^®^ (manufactured by DSM) (DHA was added at 2% total calories, and olive oil was added to maintain caloric balance). The dose of DHA, i.e., 2% total calories, is equivalent to a dose of Lovaza^®^ (4 g/day) used clinically to treat NASH (114). Mice were maintained on these diets for an additional 8 weeks. Liver and blood were collected at euthanasia. Hepatic and RBC total lipids were extracted, saponified, methylated, and quantified by gas chromatography, as described in Reference 87. In addition to the fatty acid profile, we report on the ω6/ω3 ratio for each treatment in both human and mouse liver. Asterisk denotes significantly different (<0.05) percentages from minimal findings in humans and reference diet in mice. Abbreviations: DHA, docosahexaenoic acid; NAFLD, nonalcoholic fatty liver disease; NASH, nonalcoholic steatohepatitis; PUFA, polyunsaturated fatty acid; RBC, red blood cell; WD, western diet.

To illustrate the impact of steatosis and NASH on hepatic ω3 and ω6 C_18–22_ PUFAs, we present data from human (2) and mouse studies (87). The human study reported on the fatty acid profile in livers and red blood cells (RBCs) of patients with minimal findings associated with NASH, patients with simple steatosis, and patients with NASH. Figure 3a illustrates the effect of liver health on ω6 and ω3 C_18–22_ PUFA content in human liver and RBCs. When compared with the minimal findings group, patients with NASH had a significant reduction in both arachidonic acid (ARA) (20:4, ω6) and DHA in liver. In addition, the hepatic ω6/ω3 PUFA ratio was 1.8- to 2.3-fold higher in livers from patients with simple steatosis and NASH, respectively. In contrast to liver, RBC ω3 and ω6 C_18–22_ PUFA abundance was not different among the three patient groups, ruling out systemic deficiency of ω3 and ω6 PUFAs. As such, patients with hepatosteatosis and NASH have a hepatic deficiency, but not a systemic deficiency, of C_20–22_ ω3 and ω6 PUFAs.

In a follow-up study, Arendt et al. (8) reported dysregulation of expression of key fatty acid desaturases (Fads1, Fads2) involved in generating C_20–22_ ω3 and ω6 PUFA. We should point out that the elongases (Elovl2 and Elovl5) and desaturases (Fads1 and Fads2) are used to convert both linoleic acid (LA) (18:2, ω6) and ALA to C_20–22_ PUFAs (57, 60). As such, the findings by Arendt et al. (8), coupled with the RBC data, suggest that the decline in hepatic C_20–22_ ω3 and ω6 PUFAs in NASH patients was due not to a dietary deficiency of essential fatty acids (LA and ALA) but to tissue-specific dysregulation of the pathway for dietary C_18_ ω3 and ω6 PUFA conversion to C_20–22_ ω3 and ω6 PUFAs. Because dietary ALA is typically much lower in abundance than dietary LA, the impact of impaired C_20–22_ PUFA synthesis is likely to affect hepatic levels of C_20–22_ ω3 PUFAs more than C_20–22_ ω6 PUFAs. An alternative explanation for the decline in hepatic C_20–22_ PUFAs is that liver composition changes as steatosis and fibrosis progress to cirrhosis and HCC, from predominantly hepatocytes to fibrotic tissue. According to human single-cell data from The Human Protein Atlas, hepatocytes express high levels of all of the enzymes required for essential fatty acid conversion to C_20–22_ ω3 and ω6 PUFAs, whereas other cell types (Kupffer, stellate, cholangiocyte, endothelial, T, and B cells) express these enzymes at low levels (see https://www.proteinatlas.org/). As such, dietary supplementation with C_20–22_ ω3 and ω6 PUFAs may have some benefit.

For comparison with the human study, we examined hepatic and RBC PUFA profiles from a study carried out using male *Ldlr*^−*/*−^ mice (Figure 3) (87). This study was designed to test the capacity of dietary DHA supplementation to reverse western diet (WD)-induced liver damage. We use previously published liver fatty acid data and unpublished RBC fatty acid data to generate the graphs in Figure 3b. The WD used in these studies contained 41% energy as fat (milk fat and lard), 42% energy as carbohydrate (~50% of the carbohydrate was sucrose), and 17% energy as protein, plus 0.15% w/w cholesterol (https://researchdiets.com/formulas/D12079Bi). Essential fatty acids (ALA and LA) in the WD are low, but not absent. Male *Ldlr*^−*/*−^ mice were maintained on a reference diet (Purina 5053 chow: 13.5% energy as fat, 58% energy as carbohydrate, and 28.5% energy as protein) for the 30-week duration of the study. Mice fed the WD for 22 weeks were obese and displayed markers of NAFLD, e.g., hepatosteatosis and elevated blood ALT. These mice were switched to the WD supplemented with either olive oil or DHA. DHA was added at 2% total calories, and olive oil was added to maintain caloric balance. The dose of DHA was equivalent to the clinical dose of Lovaza^®^ (4 g/day) used to treat human NASH (114). Mice were maintained on these diets for 8 weeks. At the termination of the experiment, hepatic and RBC total lipids were extracted, saponified, methylated, and quantified by gas chromatography (87). The results are expressed as fatty acid mole percent.

LA is the major PUFA in liver and RBCs, and the hepatic and RBC abundance of LA were reduced by feeding mice the WD for 22 and 30 weeks, respectively (Figure 3). Interestingly, the abundance of the elongation and desaturation product of LA, that is, ARA, was not affected by the WD. However, ARA levels in both liver and RBCs were suppressed by dietary DHA supplementation. DHA is an end product inhibitor of both ω3 and ω6 C_20–22_ PUFA synthesis; DHA suppresses the expression of Fads1 and Elovl5 (31, 60).

ALA is the major dietary source of ω3 PUFAs, but its abundance was very low in RBCs and livers of mice maintained on the reference diet for the duration of the experiment, and feeding mice the WD further suppressed hepatic ALA abundance. The major ω3 PUFA appearing in both liver and RBCs was DHA, and its hepatic and RBC abundance decreased with WD feeding. DHA supplementation of the WD increased DHA and EPA in both RBCs and liver; DHA is converted to EPA through retroconversion (Figures 2 and 3). Low hepatic EPA and DHA were associated with NASH, while increasing hepatic EPA and DHA through dietary supplementation were associated with decreased NASH severity (87). The outcome of this analysis indicates that in both humans and mice, obesity and NAFLD increase the hepatic ω6/ω3 PUFA ratio, and a high ω6/ω3 PUFA ratio is associated with increased inflammation (119). DHA supplementation of WD-fed *Ldlr*^−*/*−^ mice increased both hepatic DHA and EPA and decreased both hepatic ARA and the ω6/ω3 PUFA ratio.

Dietary ω3 and ω6 C_18–22_ PUFAs and their metabolites are substrates for neutral lipids (DAGs, TAGs, and CEs) and glycerophospholipids. The C_20–22_ PUFA products are rarely, if ever, found in sphingolipids (41). As such, the C_18–22_ PUFAs are present in cellular membranes, including the plasma, mitochondrial, peroxisomal, lysosomal, and endosomal membranes, as well as in storage vesicles, such as lipid droplets. Membrane-associated PUFAs affect membrane fluidity and lipid raft composition. Many signaling pathways rely on lipid raft composition for function (60). Moreover, fatty acyl chains can be excised from membrane lipids by phospholipases and serve as substrates for bioactive lipids (oxylipins), for example, series 2 and series 3 prostaglandins, epoxy and dihydroxy fatty acids, resolvins, and protectins. These bioactive oxylipins regulate cell function through plasma membrane G-protein and nuclear receptors, that is, the peroxisomal proliferator-activated receptor (PPAR) family (PPARα, -β, and -γ) (36, 96, 116). As indicated in the sidebar titled Why Focus on ω3 PUFAs?, C_18–20_ ω3 and ω6 PUFAs are pleiotropic regulators of multiple hepatic pathways relevant to NAFLD. As such, hepatic deficiency of ω3 PUFAs is likely to have adverse effects on overall hepatic function.

## ω3 PUFA EFFECTS ON MECHANISMS CONTRIBUTING TO NAFLD

We next describe the cellular and molecular events associated with the transition from a healthy liver to one with steatosis, inflammation, and fibrosis. We also present evidence from preclinical studies that assess the impact of dietary ω3 PUFAs on NAFLD hepatic steatosis, inflammation, and fibrosis.

### Hepatosteatosis

The accumulation of lipid in liver, reflected by hepatosteatosis, is considered to be the first hit in a multihit, multicellular process leading to NASH. Several pathways contribute to the management of hepatic lipid levels, including the uptake of NEFAs from the circulation, de novo lipogenesis (DNL), hepatic neutral lipid synthesis, fatty acid oxidation (FAO), and storage and export of very low-density lipoproteins (VLDLs). To gain insight into the sources of hepatic fat in obese NAFLD patients, Donnelly et al. (33) used a diet containing 30% fat as calories supplemented with stable tracer isotopes, that is, [^13^C_1_]-sodium acetate, [1,2,3,4 ^13^C_4_]-potassium palmitate, and [^2^H_31_]]-tripalmitin, in hypertriglyceridemic and hyperinsulinemic NAFLD patients. The key findings were that the serum NEFA pool was the primary contributor (59%) to hepatic TAG and lipoprotein TAG in VLDL in the fasting and fed states. Dietary fat contributed ~15% to both liver and VLDL-TAG (33). DNL was elevated during fasting, was a major contributor to hepatosteatosis, and was responsible for 26% of hepatic TAG and 23% of VLDL-TAG (33).

The major source of serum NEFA during fasting is adipose tissue lipid. While mobilization of lipid from adipose tissue is a normal process during fasting, problems occur when adipocytes become resistant to insulin action; insulin normally suppresses lipid mobilization. Accordingly, adipocyte insulin resistance is associated not only with increased lipid mobilization but also with increased adipocyte and systemic inflammation (46). Lipolysis of adipocyte neutral lipid, delivery of NEFA to the circulation, and NEFA uptake by hepatocytes lead to increased neutral lipid storage in liver. Since total body stores of lipid in adipose tissue increase with obesity, controlling body weight and improving insulin action are key treatment strategies for NAFLD.

As noted above, hepatic DNL represents a significant source of lipid accumulating in the livers of NAFLD patients. DNL is induced by excessive dietary carbohydrate consumption and elevated plasma insulin (59, 60). In addition, low dietary PUFAs increase hepatic DNL (43). The inhibitory effect of dietary PUFAs on DNL has been well established in preclinical models and is due to PUFA regulation of transcription factors controlling expression of proteins involved in DNL, namely, acetyl CoA carboxylase (Acc) and fatty acid synthase (Fasn) (56, 60). While insulin and dietary glucose stimulate the accumulation of sterol regulatory element binding protein 1C (Srebp1c) and the carbohydrate regulatory element binding protein/Max-like factor X (Chrebp/MLX) heterodimer in hepatic nuclei, leading to increased expression of proteins involved in DNL, dietary PUFAs suppress these events, leading to suppression of DNL. Srebp1c and Chrebp/MLX regulate the expression of multiple proteins involved in DNL, as well as enzymes involved in fatty acid remodeling, that is, desaturases and elongases. These proteins include fatty acid desaturases Fads1 and Fads2, stearoyl CoA desaturase (Scd1), fatty acid elongases (Elovl2, Elovl5, and Elovl6), and the glycolytic enzyme L-pyruvate kinase (14, 59, 60, 73, 124, 133, 136).

Palmitate (C16:0), stearate (18:0), and oleate (C18:1, ω9) represent the predominant saturated fatty acid (SFA) and monounsaturated fatty acid (MUFA) products of DNL in liver (59). The composition of dietary fat, adipose-derived NEFAs, and the activity of hepatic enzymes involved in DNL along with enzymes involved in hepatic fatty acid desaturation and elongation determine the ratio of SFAs to unsaturated fatty acids in liver and plasma (76). Increased expression of hepatic Scd1, commonly seen in preclinical models of NAFLD (41, 86), increases the production of MUFAs (C16:1, ω7; C18:1, ω7; and C18:1, ω9). While Elovl5 and Elovl6 are involved in converting C16:1, ω7 to C18:1, ω7, Elovl6 converts C16:0 to C18:0. NAFLD patients with increased hepatic palmitoleic acid (C16:1, ω7) and *cis*-vaccenic acid (18:1, ω7) reflect the involvement of Scd1 and Elovl5 in MUFA synthesis (59, 72, 76).

Additional problems associated with hepatic metabolism in NAFLD patients involve hepatic FAO, VLDL secretion, mitochondrial dysfunction, and endoplasmic reticulum and oxidative stress. Disruptions of VLDL assembly and secretion may play a role in the accumulation of hepatic lipid in some populations with NAFLD (81). For example, there is a disparity in individuals who develop NAFLD: Males are more prone to develop NASH and fibrosis than premenopausal females (66, 102, 142). Estrogen plays a protective role in preventing hepatic stellate cell (HSC) activation and fibrosis (142). A recent report suggests that the estrogen-related receptor ERRα, a nuclear receptor involved in regulating expression of genes involved in mitochondrial oxidative phosphorylation and lipid metabolism, is indispensable for VLDL synthesis and secretion and may account for the sex disparity reported in NAFLD prevalence (144).

A second factor associated with NAFLD in some populations is choline deficiency (<50% adequate intake). Choline is a key micronutrient required for one-carbon metabolism and the synthesis of phosphatidylcholine, a key membrane phospholipid in the endoplasmic reticulum, mitochondria, and VLDL (27). Guerrerio et al. (45) reported that decreased choline intake was associated with increased fibrosis in postmenopausal women, but choline intake was not associated with NAFLD in men, children, or premenopausal women. This is an example of the use of personalized dietary supplementation to mitigate NAFLD progression.

Excess NEFA entering hepatocytes has the potential to overload mitochondrial β-oxidation, resulting in accumulation of acylcarnitines, leading to incomplete FAO, impaired ketogenesis, uncoupling of mitochondrial respiration, and the generation of reactive oxygen species (ROS) within mitochondria, such as superoxide, hydrogen peroxide, hydroxy radicals, and singlet oxygen (59). These molecules have the capacity to modify cell function by changing lipid, protein, and nucleic acid structure (1, 59, 69, 128). Insulin resistance also promotes mitochondrial dysfunction arising from lipid overload and impairment of FAO pathways (25, 62, 128, 149). The formation of ROS reduces mitochondrial biogenesis and affects autophagy, mitophagy, fission, and fusion (95).

Membrane lipids contain saturated and unsaturated fatty acyl chains. The double bonds in membrane fatty acyl chains are particularly vulnerable to nonenzymatic oxidation and the formation of lipid peroxides. The presence of lipid peroxides affects membrane integrity and impairs membrane-associated metabolic processes.

The inner membrane of mitochondria, for example, is enriched in cardiolipin. Cardiolipin is a complex lipid consisting of two glycerols joined through an ester linkage and two fatty acyl chains esterified to each glycerol moiety, that is, 1,3-bis-(*sn-*3′-phosphatidyl)-*sn*-glycerol (104). Cardiolipin is interesting in the context of metabolic disease because the acyl chains in cardiolipin are predominantly C_18–22_ PUFAs, both ω3 and ω6, and diet significantly affects cardiolipin acyl chain composition and mitochondrial function (23, 78, 100, 103, 104, 106, 125, 126). Oxidation of mitochondrial matrix cardiolipin has the potential to significantly impair matrix membrane structure and function. Insufficiency of antioxidants, such as vitamin E, vitamin C, and glutathione, exacerbate mitochondrial damage.

### Nonenzymatic and Enzymatic Lipid Oxidation

ROS-induced nonenzymatic peroxidation of PUFAs is associated with NAFLD progression to NASH and the formation of lipid peroxides, including ARA-derived F2 and EPA-derived F3 isoprostanes (32). These products appear in the urine and plasma of NASH patients and the liver of mice with NASH (32). These compounds are systemic markers of oxidative damage of membrane lipids (48). Plasma LA-derived oxylipins such as 9- and 13-(±)-9-hydroxy-10E,12Z-octadecadienoic acid (HODE) are established markers of oxidative stress, and increases of 9- and 13-HODE in plasma are associated with advancing NAFLD in humans (88). Increased oxidative stress promotes cytokine [i.e., tumor necrosis factor α (TNFα)] production, further exacerbating mitochondrial stress and hepatocellular injury and leading to HSC activation and hepatic fibrosis (66). The aberrant accumulation of products from hepatic oxidative stress is associated with inflammation as NAFLD progresses to NASH.

### Enzymatically Derived Oxylipins: Friend or Foe?

In addition to nonenzymatic lipid peroxidation products, there are multiple enzymatic pathways in the liver for generating oxylipins. PUFAs can be oxidized to form oxylipins with epoxy, mono- and dihydroxy, and peroxy groups through enzymatic pathways. These pathways include the synthesis of prostaglandins, prostacyclins, thromboxanes, leukotrienes, and epoxy and dihydroxy fatty acids, as well as resolvins and protectins (36, 96, 113). Enzymatically generated oxylipins affect cell function through multiple mechanisms including G-protein-linked receptors and nuclear receptors, for example, prostaglandin DP1–2 receptors, prostaglandin EP1–4 receptors, prostaglandin F2α receptor, prostaglandin I2 receptor, thromboxane A2 receptor, and PPARα, -β, and -γ (39, 70, 96).

Hepatic oxylipins are present at very low levels requiring targeted liquid chromatography/mass spectrometry approaches for detection and quantitation. Preclinical studies have established that the WD affects hepatic content of multiple oxylipins (40, 41). Moreover, supplementation of the WD with DHA reverses many, but not all, of the WD-induced changes in oxylipins (40). Below, we briefly describe the effects of the WD on hepatic oxylipins derived from LA, ARA, EPA, and DHA.

LA is an abundant ω6 PUFA found in hepatic membrane lipids (41), and LA can be converted to 9,10-epoxyoctadecaneoic acid (9,10-EpOME) by Cyp450 epoxygenases, likely one of the Cyp2c or Cyp2J subtypes. In subsequent reactions, 9,10-EpOME is converted to 9,10-hydroxyoctadecenoic acid (9,10-DiHOME) by an epoxide hydrolase, Ephx2 (40, 41). 9,10-EpOME production is induced in neutrophils as part of the respiratory burst, an event associated with NASH progression (74). Epoxy fatty acids have short half-lives and are rapidly converted to less active or inactive dihydroxy forms by Ephx2. Preclinical studies have shown that LA and some LA-generated epoxy and dihydroxy oxylipins are low in livers of WD-fed mice, a decline that parallels hepatic LA content. Low hepatic LA is due to low levels of LA in the WD (40, 41).

ARA is a substrate for multiple hepatic oxylipins, including prostaglandins (PGE2), prostacyclins (PGI2), thromboxanes (TBXA), leukotrienes [5-, 12-, and 15-hydroxyeicosatetraenoic acids (HETEs)], and epoxy fatty acids. The enzymes involved in the production of these ARA-derived oxylipins include prostaglandin synthases; thromboxane synthase and the 5-, 12-, and 15-lipoxygenases; and Cyp2c and Cyp2J. The products of these pathways regulate cell function through receptor-mediated mechanisms, as described above. Like the LA-derived oxylipins, these products are short-lived and do not accumulated in liver. As such, a measure of their production is achieved through measuring the active product and the more stable inactive metabolite.

Liver cells express prostaglandin endoperoxide synthases 1 and 2 (*Ptgs1* and *Ptgs2*), thromboxane synthase and the 5-, 12-, and 15-lipoxygenases, as well as several cytochrome P450 subtypes (*Cyp2c* and *Cyp2J*). Hepatic ARA-derived products include prostaglandins (6-keto PGF1α, PGD2, PGE2, and PGF2α), thromboxane (TBXB2), leukotrienes (5-, 12-, and 15-HETEs), and several epoxyeicosatrienoic acids (e.g., 11,12-EpETrE) and dihydroxyeicosatrienoic acids (e.g., 11,12-DiHETrE). The active forms of these oxylipins are thought to regulate hepatic inflammation, metabolism, and blood flow (39).

EPA-derived products include multiple epoxy-eicosatetraenoic acids (EpETEs), dihydroxyeicosatetraenoic acids (DiHETEs), and resolvin E1 (RVEve1), while DHA-derived products include several epoxydocosapentaenoic acids (EpDPEs) and dihydroxydocosapentaenoic acids (DiHDPEs), as well as resolvin D1 (RVD1) and protectin X (PDX) (40, 41).

In mice, WD feeding lowers hepatic C_18–22_ ω3 and LA but not ARA (Figure 3) (87). Hepatic levels of oxylipins derived from these precursors parallel the hepatic abundance of the precursors, LA and ARA (40). Addition of DHA to the WD, however, effectively increases both hepatic EPA and DHA and the EPA- and DHA-derived epoxy and dihydroxy oxylipins. Moreover, dietary DHA lowers hepatic levels of ARA as well as the ARA-derived oxylipins; DHA inhibits the conversion of LA to ARA (40). It is generally viewed that oxylipins derived from ARA are proinflammatory, while oxylipins derived from EPA and DHA are anti-inflammatory (36) and hepatoprotective (40). Thus, hepatic content of C_20–22_ ω3 PUFAs plays an important role in controlling hepatic levels of putative pro- and anti-inflammatory oxylipins.

While ω3 PUFA-derived oxylipins are associated with hepatoprotection (31, 40), the molecular basis for this protection is not fully established. To gain some insight into this process, Schuck et al. (113) used wild-type C57BL/6J and *Ephx2*^−*/*−^ mice on the C57BL/6J background and reported that feeding an atherogenic diet induced hepatic injury and inflammation in wild-type mice. *Ephx2*^−*/*−^ mice fed the same diet, however, had a significant reduction in hepatic pathology. These and other studies, reviewed by Morisseau & Hammock (96), support the notion that the epoxy fatty acids are hepatoprotective, while the dihydroxy fatty acids are less active or inactive metabolites. Our preclinical studies suggest that changes in hepatic ω3 PUFA-derived epoxy and dihydroxy oxylipins are driven more by substrate abundance rather than by large changes in the expression of the various Cyp2 and Ephx subtypes involved in epoxy and dihydroxy oxylipin synthesis (40).

These preclinical studies establish that liver has the capacity to generate a large number of distinct oxylipins from ω3 and ω6 PUFAs. Moreover, hepatic precursor levels are major determinants of the hepatic abundance of these oxylipins. As such, decreasing the hepatic ω6/ω3 PUFA ratio by increasing dietary C_20–22_ ω3 PUFA supplementation increases production of ω3 PUFA-derived oxylipins and decreases production of ω6 PUFA-derived oxylipins (40, 87). These changes in the hepatic ω6/ω3 PUFA ratio and oxylipin type are associated with hepatoprotection against diet-induced NAFLD. While it is clear that the liver has the capacity to generate multiple oxylipins from LA, ARA, EPA, and DHA, the specific molecular targets of these oxylipins and their effects on hepatic function remain to be established.

### NAFLD, Inflammation, and the Gut–Liver Axis

A key question in the NAFLD field deals with the signals that initiate the transition from macrosteatosis to NASH. An emerging theme to account for this transition involves gut dysbiosis and its effects on the liver, that is, the gut–liver axis (10, 79, 120, 134). The liver is the first organ to encounter dietary products absorbed by the gut (134). Moreover, there is a bidirectional flow of compounds between the gut and liver. Products absorbed by the gut and entering the hepatic portal circulation include water-soluble micro- and macronutrients, secondary bile acids, and microbial metabolites, while hydrophobic compounds (fats, sterols, and fat-soluble vitamins) are first transferred to the lymphatics as chylomicron and delivered to the systemic circulation at the level of the thoracic arch. The liver is a source of bile acids, and these primary bile acids are delivered to the gut via the hepatopancreatic duct. Gut microbes convert primary bile acids to secondary bile acids, which are returned to the liver via enterohepatic circulation (138).

In addition to micro- and macronutrient absorption, gut-derived products also include microbial components. Some microbial components are used by intestinal cells for metabolism, such as butyrate for colonic metabolism, while other microbial products have adverse effects on liver cells, as well as systemically. Endotoxinemia, for example, is commonly associated with NASH (49, 64, 93). The appearance of gut-derived microbial products in the circulation can arise from failure of gut barrier integrity where tight junctions between enterocytes are impaired (leaky), leading to increased permeability and the appearance of gut luminal products in the circulation (15, 63). Alternatively, some hydrophobic microbial components are coabsorbed with lipids during chylomicron assembly and are transported to the lymph and then to the circulation (44).

The level of endotoxin in NASH patients, while elevated, is low when compared with blood levels of endotoxin seen in sepsis (49). As such, NASH is associated with a low-grade systemic metabolic endotoxinemia (21, 93). Endotoxinemia is also a common feature associated with NAFLD in preclinical models of obesity and insulin resistance (31, 93). For example, Cani et al. (21) reported that metabolic endotoxinemia initiates obesity and insulin resistance, while Spruss et al. (123) reported that Toll-like receptor 4 (TLR4) is involved in fructose-induced insulin resistance in mice.

Endotoxin, also known as lipopolysaccharide (LPS), is a cell-wall component of gram-negative bacteria. Endotoxin is only one of many gut-derived microbial products appearing in the circulation. These products are referred to as pathogen-associated molecular patterns (PAMPs), and PAMPs activate TLRs. A second source of inflammatory factors activating TLRs are products from dead and dying cells, also known as damage-associated molecular patterns (DAMPs). Together, PAMPs and DAMPs activate the innate immune system through TLRs and other pattern recognition receptors.

Hepatic resident macrophages, that is, Kupffer cells, express receptors for multiple plasma membrane and endosomal TLRs, including TLR1, TLR2, TLR4, TLR5, TLR6, and TLR9 (134). As such, Kupffer cells have the capacity to respond rapidly to changes in circulating PAMPs. Each TLR responds to a unique set of agonists. For example, endotoxin is a TLR4 agonist, while the TLR2 agonist lipoteichoic acid is from the cell wall of gram-positive bacteria. The general scheme for TLR2 activation of macrophages involves TLR2 heterodimerization with TLR6 or TLR1 and the interaction of the TLR2 heterodimer with Cd14. Cd14 functions as a ligand carrier (e.g., lipoteichoic acid) and provides delivery to the TLR2 heterodimer. Liganded TLR2 initiates a cell signaling cascade mediated by MyD88 and MyD88 adapter-like (Mal)/TIR-domain-containing adapter protein (TIRAP). Endotoxin uses a similar scheme including Cd14 and Myd88, as well as additional protein mediators, such as LPS-binding protein (LPSBP), TLR4, and MD2. Endotoxin activation of this pathway also targets nuclear factor kappa B (NFκB) signaling. Ligand activation of both TLR2 and TLR4 stimulates the expression of chemokines, for example, monocyte chemoattractant protein-1 (Mcp1), and proinflammatory cytokines, for example, interleukins (IL6 and IL1β) (134), that promote hepatic infiltration of immune cells, including monocytes, macrophages, B cells, and T cells, as well as the activation of repair processes and ECM remodeling (59, 60, 67, 109).

Using a preclinical model of NASH, we established that plasma levels of both TLR2 and TLR4 agonists increase in response to the WD (32, 85). TLR2, but not TLR4, agonist levels in plasma are reduced with DHA dietary supplementation (85, 87). Systemic markers of inflammation, such as plasma TNFα and microbial factors, for example, TLR2 agonist, also precede the appearance of macrosteatosis and fibrosis, supporting the notion that diet-induced gut dysbiosis, resulting in the production of systemic inflammatory factors, is an early event in the onset and progression of liver disease to NASH.

Additional support for the role of gut microbiota in hepatic inflammation and NASH is found in the following studies. Hepatosteatosis does not develop in germ-free mice fed a western-like diet (61). Moreover, abrogation of TLR2 (92, 141), TLR4 (123), or Myd88 (35) function by gene deletion or antibody neutralization protects mice from diet-induced NASH. Thus, gut microbiota play a major role in NAFLD onset and progression, involving both TLR2 and TLR4 pathways and likely other TLR pathways in NASH pathogenesis.

DHA supplementation of the WD attenuates the expression of multiple components of TLR signaling including the expression of TLR2 and TLR4 receptors and Cd14 and NFκB-p50 nuclear abundance (31, 85, 87). DHA effects on these proteins are seen in both prevention and remission studies of WD-induced NASH (31, 85, 87). Further evidence for PUFA regulation of TLRs involved studies on TLR dimerization within membranes. While SFAs stimulated TLR2-TLR1 heterodimerization, a requirement for the initiation of cell signaling, PUFAs inhibited dimerization (77). TLR2 and TLR4 function within lipid rafts on the apical surfaces of cells in association with key proteins such as caveolin-1 (118, 121). While SFAs, cholesterol, and sphingomyelin stabilize lipid rafts, DHA disrupts lipid raft formation (24). However, dietary DHA does not lower WD-induced TLR4 agonists in plasma (32, 85, 87). The outcomes of these and other studies indicate that DHA can lower hepatic expression of TLR receptors and Cd14 potentially lower TLR2 agonists, disrupt TLR signaling at the plasma membrane, and lower nuclear NFκB-p50 levels (31, 85). Clearly, DHA has the capacity to reduce the impact of gut dysbiosis on the liver.

### Hepatic Injury and Fibrosis

Diet-induced hepatocellular death stimulates HSCs and the production of proteins involved in fibrosis, such as Col1a1, Acta2, and Eln (54). HSCs are located in the space of Disse, between sinusoidal endothelial cells and hepatocytes. HSCs account for 5–8% of the hepatic cell population and harbor the largest reservoir of vitamin A in the human body (145). Normally HSCs are quiescent, but following hepatic injury, HSCs are activated to a promyofibroblastic phenotype where they decrease their vitamin A content, proliferate, and produce various ECM proteins (12). Several growth factors are involved in initiating and sustaining proliferation of these cells, including the transforming growth factor β (TGFβ) family, epidermal growth factor (EGF) family, connective tissue growth factor, and platelet-derived growth factors (112, 139). With acute liver injury, parenchymal and nonparenchymal cells release factors activating HSCs to produce ECM components, forming a temporary scar, and secretion of factors promoting the regeneration of hepatic epithelial cells (145).

According to single-cell RNA sequencing (RNASeq) analyses, human hepatocytes, leukocytes, Kupffer cells, stellate cells, cholangiocytes, and endothelial cells express genes encoding MMPs and TIMPs (see https://www.proteinatlas.org/). Under normal conditions of tissue repair, MMP expression increases while TIMP expression decreases, and the interaction of these two enzyme families favors ECM removal (13). This is a homeostatic mechanism guarding against prolonged generation of scar tissue resulting from injury. Chronic diseases such as NASH, however, result in persistent HSC activation and induction of multiple MMP and TIMP subtypes, resulting in excessive ECM deposition (80). In mice, WD feeding increases the expression of multiple MMPs, TIMPs, Col1a1, Eln, Acta2, collagen cross-linking enzymes (e.g., lysyl oxidases and lysyl oxidase-like subtypes), and protease inhibitors (e.g., plasminogen activator inhibitor 1) (59, 85).

Like inflammation, peripheral tissues and diverse cell types contribute to the regulation of hepatic fibrosis (85). Plasma TNFα and gut-derived TLR agonists, adipose-derived leptin, TGFβ-secreting stellate cells, macrophages, and T cells are involved in promoting hepatic fibrosis (12, 85). Further, endotoxin-activated NFκB in HSCs perpetuates inflammatory NFκB-dependent gene expression that enhances the profibrogenic response to TGFβ in HSCs (12).

A key factor in hepatic fibrosis is TGFβ. Both mice and humans express three TGFβ subtypes, and these subtypes are expressed in most hepatic cell types, including hepatocytes, Kupffer cells, stellate cells, endothelial cells, cholangiocytes, T cells, and B cells (see https://www.proteinatlas.org/). According to single-cell RNASeq analysis, all of the above-listed hepatic cells have the capacity to express at least one of the three TGFβ subtypes (TGFβ1–3) (85). Recent studies show that TGFβ2 and TGFβ3 play major roles in hepatic fibrogenesis (34, 127).

TGFβ binds cell-surface TGFβ receptor subtypes (TGFβR1–3), which mediate changes in cell function through canonical and noncanonical pathways (6, 59, 90). The canonical pathway involves TGFβ binding to its receptors, TGFβR1 and TGFβR2, which increased expression of a transcription factor, that is, Smad (a homolog of the drosophila protein, mothers against decapentaplegic, and the *Caenorhabditis elegans* protein for small body size). Activation of TGFβ signaling stimulates recruitment and phosphorylation of Smad2 and Smad3 (6). Phospho-Smads are translocated to nuclei where they bind promoters and induce transcription of genes encoding Col1a1, PAI1, cMyc, and Smad7 (an inhibitor of TGFβ signaling) (6, 12, 59). The noncanonical TGFβ signaling pathway involves TGFβR activation and the regulation of the EGF receptor and activation of RAS/MapK, TGFβ-activated kinase (Tak1), PI3K, and AKT pathways to increase expression of fibrogenic genes (6, 12, 59). TGFβ is a major component in NAFLD progression to NASH, as evidenced by Kupffer cell expression and secretion of TGFβ, an event associated with hepatic inflammation and HSC activation leading to ECM deposition and overt fibrosis. Hepatic fibrosis severity increases the risk of progressing to cirrhosis and HCC, and HCC is the chief predictor of mortality for the NAFLD patient (129).

The WD induces hepatic fibrosis (31, 41, 85–87), and DHA supplementation attenuates WD-induced hepatic fibrosis in mice. Depner et al. (31) assessed the impact of EPA, DHA, and the combination of EPA plus DHA on WD-induced NASH and fibrosis in male *Ldlr*^−*/*−^ mice. The study provided the first evidence that ω3 PUFAs have the capacity to regulate expression of genes encoding proteins associated with hepatic fibrosis, such as Col1a1. A follow-up study on the same tissue samples using a microarray approach provided an expanded view of WD and ω3 PUFA effects on hepatic gene expression linked to fibrosis. Lytle et al. (85) established that the WD increased hepatic expression of multiple TGFβ and TGFβR subtypes, collagens (e.g., Col1A1 and Col1A2), TIMPs (TIMP1–3), MMPs (MMP-2, −12, and −13), and lysyl oxidase (Lox) and lysyl oxidase-like enzymes (LoxL). DHA was more effective than EPA at suppressing expression of these transcripts (41, 85). This comparative study provided preclinical evidence that DHA was superior to EPA in decreasing hepatosteatosis, inflammation, and fibrosis and was effective in NASH prevention (31).

## CURRENT THERAPEUTIC STRATEGIES FOR NAFLD

Since there are no FDA-approved therapies for NAFLD and NASH (122), current treatment strategies focus on the comorbidities associated with the disease, such as obesity and diabetes. NAFLD diagnosis is based on assessing hepatosteatosis, inflammation, and fibrosis by histology scoring as well as imaging methods and plasma biomarkers of liver injury, for example, ALT, AST, and GGT, as described above. NAFLD diagnosis also includes an assessment of alcohol consumption and that no other causes of steatosis are present, such as viral hepatitis (hepatitis B and C viruses), drugs, or other diseases linked to fatty liver, for example, hemochromatosis, thyroid autoimmunity, Wilson’s disease, and α1 antitrypsin deficiency (89).

Lifestyle modifications including weight loss, dietary modification, and treatment of underlying MetS features are the current approaches for NASH therapy (107, 147). A number of diets have been assessed for the capacity to reduce body weight and treat NAFLD, including the low-carbohydrate, ketogenic, high-protein, Mediterranean, paleolithic, and plant-based diets, as well as intermittent fasting. Of these, the Mediterranean diet has received the most attention, with some promising results (94).

Probiotics and/or prebiotics have also been used as a NAFLD treatment strategy. Recent meta-analyses (82, 117) concluded that the use of pro- and prebiotics improved liver-specific markers in NAFLD patients. However, this approach is in an early stage of development, and more study is needed to identify specific probiotics and prebiotics that restore gut health.

A third approach uses bariatric surgery to reduce body weight in the morbidly obese patient. A recent review of the value of bariatric surgery for the NASH patient had the objective of investigating the long-term relationship between bariatric surgery, major adverse liver outcomes, and major adverse cardiovascular events in obese patients with biopsy-proven fibrotic NASH without cirrhosis (3). The study included 1,158 patients (both males and females) with a mean age of 49.8 years and a mean BMI of 44.1. Bariatric surgery was associated with a significant reduction in the risk of major adverse liver outcomes when compared with patients receiving nonsurgical disease management (3).

Pharmaceutical approaches that focus on NAFLD comorbidities, specifically diabetes, insulin resistance, and obesity, have the goal of controlling blood glucose, improving insulin action, and decreasing body weight. These treatments include metformin (an AMPK activator); inhibitors of renal glucose reabsorption [sodium-glucose cotransporter 2 (Sglt2)] (59, 122); improvement of insulin signaling, for example, glucagon-like peptide 1 (Glp1) receptor agonists such as semaglutide (98); and PPARγ agonists, for example, pioglitazone (71).

The current recommendations of the American Association for the Study of Liver Disease include targeting insulin resistance using a PPARγ agonist in combination with vitamin E for adult NASH patients, while only vitamin E is recommended for children (22, 122). A recent report by Rowe et al. (110) summarized the status of pharmaceutical approaches for NAFLD. In addition to those already mentioned, other approaches include PPARα and PPARδ agonists, thyroid hormone receptor β agonist, inhibition of chemokine receptors 2 and 5, DNL, and the mitochondrial pyruvate carrier. These approaches are currently in clinical trials.

## EFFICACY OF ω3 PUFAs IN NAFLD THERAPY

The diet most commonly associated with NAFLD is a western-like diet—a diet high in saturated fats, monounsaturated fats, trans fats, cholesterol, and simple sugars; low in fiber and ω3 and ω6 PUFAs; and with a high ω6/ω3 PUFA ratio (59). As described above, patients with NASH, cirrhosis, and end-stage liver disease have low hepatic PUFA levels (2, 7–9, 17–19, 26, 42). This hepatic deficiency of C_20–22_ ω3 PUFAs can be corrected with dietary C_20–22_ ω3 PUFA supplementation. Both clinical and preclinical studies established that dietary supplementation with C_20–22_ ω3 PUFAs increases RBC and hepatic levels of these fatty acids (87, 115). As such, attention to essential fatty acid sufficiency and dietary supplementation with C_20–22_ ω3 PUFAs should be a component of NAFLD therapy.

We recently reviewed the literature on the use of C_20–22_ ω3 PUFA dietary supplementation to treat NAFLD in both children and adults (59, 122). The rationale for the use of C_20–22_ ω3 PUFAs in NAFLD therapy is based on the well-established role these fatty acids play in the control of signaling pathways that (*a*) suppress DNL, that is, Srebp1c and Chrebp/MLX; (*b*) induce FAO, that is, PPARα (58, 60); (*c*) promote triglyceride catabolism (135); and (*d*) attenuate inflammation by suppressing NFκB signaling (20, 36) (Figure 2).

For this review, we examined registered clinical trials listed in ClinicalTrials.gov and summarized two recent meta-analyses of clinical trials involving adults and children (75, 97). Of the 33 registered clinical trials using ω3 PUFAs to treat NAFLD, only three trials have published results (30, 111, 114). Of these, only the WELCOME study (114) described an improvement in NAFLD status after treatment with pharmaceutical grade ω3 PUFA (Lovaza^®^, a mix of EPA and DHA ethyl esters). Lovaza^®^ was given at 4 g/day for 15–18 months to patients with histologically or MRI-confirmed NAFLD. When compared with the patients receiving a placebo, Lovaza^®^-treated patients had elevated blood levels of EPA and DHA and enrichment of RBCs with C_20–22_ ω3 PUFAs, decreased plasma triglycerides, and decreased hepatosteatosis, but no improvement in hepatic fibrosis (114).

Since our previous review of clinical trials and meta-analyses for children and adults with NAFLD and treatment with ω3 PUFAs (59, 122), two meta-analyses of ω3 PUFA treatment of NAFLD in children and adults have been published (75, 97). We summarize the meta-analysis by Musa-Veloso et al. (97) in Table 1. The meta-analysis included plasma (ALT, AST, and GGT) and liver markers (steatosis, hepatocellular ballooning, inflammation, fibrosis, and an overall NASH activity score) of disease. The publication dates for the trials ranged from 2005–2018, and 4–16 published studies were assessed for each parameter. The analysis included both children (<18 years of age) and adults using EPA, DHA, or both fatty acids for treatment. The dose ranged from 0.35 to 6.8 g of ω3 PUFAs/day for 2–24 months.

In Table 1 we present the difference in means for each parameter assessed, the upper and lower 95% confidence intervals, and p-values. The key issue is whether treatment had a significant effect (p-value ≤ 0.05) on a specific parameter. Of the ten parameters reported, four were significantly affected by treatment, including plasma ALT, GGT, liver steatosis as measured by magnetic resonance spectroscopy (MRS), and ultrasonography. The meta-analysis reported by Lee et al. (75) essentially confirmed the Musa-Veloso et al. (97) meta-analysis and provided additional data on plasma parameters. Specifically, dietary ω3 PUFA supplementation improved plasma triglycerides, total cholesterol, and high-density lipoprotein-cholesterol, but had no effect on hepatic fibrosis, plasma low-density lipoprotein-cholesterol, blood glucose, insulin resistance (using the homeostatic model assessment of insulin resistance), or BMI.

While these results are encouraging, there are a couple of caveats that should be mentioned. The lack of histological confirmation of steatosis and fibrosis is a common omission in many clinical studies using ω3 PUFAs to treat NAFLD. Also, the ω3 PUFAs were used alone and not in association with other therapies reported to lower the comorbidities associated with NAFLD. While MRS or ultrasound approaches are noninvasive, these approaches do not distinguish between MIS and MAS and provide no information on hepatic inflammation or details on fibrosis.

The failure of ω3 PUFA supplementation to reduce hepatic fibrosis is not surprising. As mentioned above, our preclinical studies indicate that DHA does not totally prevent diet-induced fibrosis, but DHA does reduce disease severity, including the level of hepatic fibrosis. The effect of DHA is more impressive on the expression of genes involved in fibrosis, for example, Col1A1 and Col1A2, than on fibrosis measured by histological staining, for example, picosirius red or trichrome staining (86, 87). We speculate that the failure of DHA to promote removal of preexisting fibrosis/ECM is linked to DHA suppression of expression of multiple ECM remodeling enzymes, such as MMPs, TIMPs, Lox, and LoxL. DHA treatment of *Ldlr*^−*/*−^ mice in either prevention or remission studies show that DHA effectively lowers expression of collagens, MMPs, TIMPs, Lox, and LoxL transcripts but does not fully abolish all histological evidence of fibrosis (87). As described by others (13), ECM removal requires elevated expression of MMPs and suppression of TIMPs, collagens, Lox, and LoxL transcripts.

A modification of ω3 PUFA therapy for NAFLD includes cotreatment with probiotics. The rationale for this approach is based on the well-established role that probiotics play in gut health and on the role that the gut–liver axis plays in the onset and progression of NAFLD. Interestingly, some studies in humans and mice have demonstrated that using ω3 PUFAs and probiotics together results in better outcomes in the treatment of metabolic disease than when they are used separately (68, 105, 108). Some clinical studies report improvement in insulin sensitivity, inflammatory markers, and gut microbiome diversity following ω3 PUFA plus probiotic supplementation (28, 99). Certainly, the use of pro- and prebiotics is a reasonable approach to restore gut health and should be considered as part of any NAFLD/NASH therapy.

## CONCLUSIONS

We have provided an update on the use of C_20–22_ ω3 PUFAs in human NAFLD therapy. We have also presented data from preclinical studies that reveal the strengths and limitations of using C_20–22_ ω3 PUFAs in the prevention and treatment of diet-induced NASH. According to the clinical and preclinical evidence, dietary C_20–22_ ω3 PUFA supplementation decreases human NAFLD severity by reducing hepatosteatosis and liver injury as measured by plasma ALT and GGT (Table 1). It is abundantly clear, however, that dietary C_20–22_ ω3 PUFA supplementation when used alone does not resolve all clinical features associated with NASH. Moreover, none of the targeted pharmaceutical approaches described above can resolve the hepatic C_20–22_ ω3 PUFA deficiency reported in NASH and cirrhotic patients (2, 8, 17–19). Since C_20–22_ ω3 PUFAs are pleiotropic regulators of hepatic function, the addition of dietary C_20–22_ ω3 PUFA supplementation to targeted pharmaceutical therapies may improve clinical outcomes. In addition, EPA and DHA have both independent and overlapping effects on cell hepatic function. While EPA is a more robust activator of hepatic PPARα and PPARα signaling than DHA, DHA is a more effective inhibitor of Srebp1c-mediated suppression of DNL, fatty acid elongation, and desaturation (60). Since DHA is readily retroconverted to EPA (31), we suggest that DHA is a better choice than EPA for use in mitigating NAFLD onset and progression.

## Figures and Tables

**Table 1:** Summary of meta-analysis of co3 PUFA use in NAFLD therapy (97)

Parameter	Dates of publication	Number of studies	Population	Treatment (EPA, DHA, or both)	Dose (g/day)	Duration (months)	Difference in means	95% CI lower limit	95% CI upper limit	P-value
**ALT**	2008–2016	16	Children and adults	EPA, DHA, both	0.35–4.5	3–12	−4.6	−9.2	0.08	0.046
**AST**	2006–2018	12	Children and adults	Both	0.35–4.7	3–12	−2.4	−7.3	2.5	0.34
**GGT**	2006–2018	8	Children and adults	Both	0.35–4.9	3–12	−5.6	−9.6	−1.5	0.007
Liver fat, MRS	2005–2015	5	Children and adults	DHA, both	0.25–6.8	2–12	−6.19	−9.58	−0.79	0.02
Liver fat, ultrasonography	2006–2015	7	Children and adults	DHA, both	0.25–4.7	6–24	−0.71	−0.989	−0.42	<1.001
Liver fat, histology	2015–2016	4	Adults	Both	0.35–3.6	6–24	−0.093	−0.595	−0.41	0.717
Hepatocellular ballooning	2015–2016	4	Adults	Both	0.35–3.6	6–12	−0.175	−0.052	0.271	0.443
Lobular inflammation	2015–2016	3	Adults	Both	0.345–3.6	6–24	−0.031	−0.577	0.515	0.911
Fibrosis score	2015–2016	4	Adults	Both	0.345–3.6	6–12	−0.23	−0.56	0.093	0.162
NASH activity score	2015–2016	3	Adults	Both	0.345–3.6	6–12	0.558	−0.116	1.131	0.105

Abbreviations: ALT, alanine amino transferase; AST, aspartate amino transferase; CI, confidence interval; DHA, docosahexaenoic acid; EPA, eicosapentaenoic acid; GGT, γ-glutamyl transferase; MRS, magnetic resonance spectroscopy; NAFLD, nonalcoholic fatty liver disease; NASH, nonalcoholic steatohepatitis; PUFA, polyunsaturated fatty acid.

## WHY FOCUS ON ω3 PUFAs?

1. ω3 PUFAs lower blood triglycerides.
2. ω3 PUFAs suppress de novo lipogenesis and suppress Srebp1c and Chrebp/MLX function.
3. ω3 PUFAs increase mitochondrial, peroxisomal, and microsomal fatty acid oxidation (ligands for peroxisomal proliferator-activated receptors PPARα, -δ, and -γ).
4. ω3 PUFAs are anti-inflammatory; they decrease NFκB p50, plasma TLR2 agonists, and hepatic expression of CD14, TLR2, and TLR4.
5. ω3 PUFAs are antifibrotic; they decrease western diet–induced fibrosis and suppress hepatic expression of collagens, MMPs, and TIMPs.
6. NASH patients have low hepatic ω3 PUFAs.
